# Age dependency of body mass index distribution in childhood and adolescent inpatients with anorexia nervosa with a focus on DSM-5 and ICD-11 weight criteria and severity specifiers

**DOI:** 10.1007/s00787-020-01595-4

**Published:** 2020-07-14

**Authors:** Christian Engelhardt, Manuel Föcker, Katharina Bühren, Brigitte Dahmen, Katja Becker, Linda Weber, Christoph U. Correll, Karin Maria Egberts, Stefan Ehrlich, Veit Roessner, Christian Fleischhaker, Alexander von Gontard, Freia Hahn, Ekkehart Jenetzky, Michael Kaess, Tanja Legenbauer, Tobias J. Renner, Ulrike M. E. Schulze, Judith Sinzig, Ida Wessing, Gisela Antony, Beate Herpertz-Dahlmann, Triinu Peters, Johannes Hebebrand

**Affiliations:** 1 Department of Child and Adolescent Psychiatry, Psychotherapy and Psychosomatics, University Hospital Essen (AöR), University of Duisburg-Essen, Wickenburgstrasse 21, 45147 Essen, Germany; 2 Department of Child and Adolescent Psychiatry, University Hospital Muenster, Schmeddingstraße 50, 48149 Münster, Germany; 3 Department of Child and Adolescent Psychiatry, Psychosomatics and Psychotherapy, University Hospital, RWTH Aachen, Neuenhofer Weg 21, 52074 Aachen, Germany; 4 Department for Child and Adolescent Psychiatry, Psychosomatics and Psychotherapy, Faculty of Medicine, Phillips-University and University Hospital Marburg, Hans-Sachs-Str. 6, 35039 Marburg, Germany; 5 Center for Mind, Brain and Behavior (CMBB), University of Marburg and Justus Liebig University Giessen, Giessen, Germany; 6 Department of Child and Adolescent Psychiatry, Charité Universitätsmedizin Berlin, Campus Virchow, Augustenburger Platz 1, 13353 Berlin, Germany; 7 Department of Psychiatry and Molecular Medicine, Zucker School of Medicine at Hofstra/Northwell, Hempstead, NY USA; 8 Department of Psychiatry, The Zucker Hillside Hospital, Northwell Health, Glen Oaks, NY USA; 9 Department of Child and Adolescent Psychiatry, Psychosomatics and Psychotherapy, Centre for Mental Health, University Hospital of Wuerzburg, Margarete-Höppel-Platz 1, 97080 Würzburg, Germany; 10 Division of Psychological and Social Medicine and Developmental Neurosciences, Faculty of Medicine, Technische Universität Dresden, Dresden, Germany; 11 Translational Developmental Neuroscience Section, Eating Disorder Research and Treatment Center, Department of Child and Adolescent Psychiatry, Faculty of Medicine, Technische Universität Dresden, Fetscherstraße 74, 01307 Dresden, Germany; 12 Department of Child and Adolescent Psychiatry and Psychotherapy, University Freiburg, Hauptstraße 8, 79104 Freiburg, Germany; 13 Department of Child and Adolescent Psychiatry, Saarland University Hospital, Kirrberger Straße 1, 66421 Homburg, Germany; 14 Department of Child and Adolescent Psychiatry and Psychotherapy, LVR-Klinik Viersen, Horionstr. 14, 41749 Viersen, Germany; 15 Department of Child and Adolescent Psychiatry Und Psychotherapy, University Medical Center of the Johannes Gutenberg University Mainz, Langenbeckstraße 1, 55131 Mainz, Germany; 16 Faculty of Health, School of Medicine, Witten/Herdecke University, Alfred-Herrhausen-Straße 50, 58448 Witten, Germany; 17 University Hospital of Child and Adolescent Psychiatry and Psychotherapy, University of Bern, Bolligenstrasse 111, Stöckli, 3000 Bern 60, Switzerland; 18 Section for Translational Psychobiology, Department of Child and Adolescent Psychiatry, Center for Psychosocial Medicine, University Hospital Heidelberg, Heidelberg, Germany; 19 LWL University Hospital Hamm for Child and Adolescent Psychiatry, Psychotherapy and Psychosomatics, Ruhr University-Bochum, Heithofer Allee 64, 59071 Hamm, Germany; 20 Department of Child and Adolescent Psychiatry, Psychosomatics and Psychotherapy, University of Tübingen, Osianderstraße 14-16, 72076 Tübingen, Germany; 21 Department of Child and Adolescent Psychiatry/Psychotherapy, University Hospital Ulm, University of Ulm, Steinhövelstraße 5, 89075 Ulm, Germany; 22 Department of Child and Adolescent Psychiatry, LVR-Klinik Bonn, Kaiser-Karl-Ring 20, 53111 Bonn, Germany; 23 Institute for Biomagnetism and Biosignalanalysis, University Hospital Münster, Malmedyweg 15, 48149 Münster, Germany; 24 Central Information Office, CIO Marburg GmbH, Struthweg 1, 35112 Fronhausen, Germany

**Keywords:** BMI-centile, BMI-SDS, Weight criterion, Early onset anorexia nervosa, Atypical anorexia nervosa

## Abstract

Both DSM-5 and ICD-11 have provided weight cut-offs and severity specifiers for the diagnosis of anorexia nervosa (AN) in childhood, adolescence and adulthood. The aims of the current study focusing on inpatients aged < 19 years were to assess (1) the relationship between age and body mass index (BMI; kg/m^2^), BMI-centiles, BMI-standard deviation scores (BMI-SDS) and body height-SDS at referral, (2) the percentages of patients fulfilling the DSM-5 and ICD-11 weight criteria and severity categories for AN, and (3) the validity of the AN severity specifiers via analysis of both weight related data at discharge and inpatient treatment duration. The German Registry for Anorexia Nervosa encompassed complete data sets for 469 female patients (mean age = 15.2 years; range 8.9–18.9 years) with a diagnosis of AN (*n* = 404) or atypical AN (*n* = 65), who were ascertained at 16 German child and adolescent psychiatric hospitals. BMI at referral increased up to age 15 to subsequently plateau. Approximately one tenth of all patients with AN had a BMI above the fifth centile. The ICD-11 specifier based on a BMI-centile of 0.3 for childhood and adolescent AN entailed two equally sized groups of patients. Discharge data revealed limited validity of the specifiers. Height-SDS was not correlated with age thus stunting had no impact on our data. We corroborate the evidence to use the tenth instead of the fifth BMI-centile as the weight criterion in children and adolescents. Weight criteria should not entail major diagnostic shifts during the transition from adolescence to adulthood. The severity specifiers based on BMI or BMI-centiles do not seem to have substantial clinical validity.

**Electronic supplementary material:**

The online version of this article (10.1007/s00787-020-01595-4) contains supplementary material, which is available to authorized users.

## Introduction

Underweight, fear of weight gain, and body image disturbances represent cardinal features of anorexia nervosa (AN) [1]. The definition of the underweight associated with AN has been subject to change. Thus, DSM-5 rephrased the DSM-IV TR weight criterion for AN to “a significantly low body weight in the context of age, sex, developmental trajectory, and physical health” [1]. Whereas no cut-off is provided in the A criterion, the main text specifically states that the body mass index (BMI; kg/m^2^) is a “useful measure to assess body weight for height” [1]. Based on the definition of underweight according to both the Centers for Disease Control and Prevention (CDC) and the World Health Organization (WHO) a BMI ≤ 18.5 kg/m^2^ was provided as a guideline for adults [1]. However, a BMI above 18.5 kg/m^2^ “might be considered to have significantly low weight if clinical history or other physiological information supports this judgement” [1]. For childhood and adolescent patients, DSM-5 refers to the CDC definition of underweight based on a “BMI-for-age below the fifth centile” [1]. The main text cautiously states that clinicians need to consider “available numerical guidelines, as well as the individual’s body build, weight history, and any physiological disturbance” [1] in judging the weight of a young patient; somewhat higher cut-offs may be appropriate for individual patients. Overall, BMI-centiles were introduced to allow comparability of body weight adjusted for height across childhood and adolescence in light of the age dependency of absolute BMI-values; Hebebrand and coworkers had initially suggested replacement of the DSM-IV weight criterion (body weight less than 85% of that expected) with the 10th BMI-centile in 1996 [2].

The more recent ICD-11 [3] weight criterion is shorter and provides a strict cut-off. Specifically, “AN is characterized by significantly low body weight for the individual’s height, age and developmental stage” [3]; the diagnosis is dependent on a “BMI less than 18.5 kg/m^2^ in adults and BMI-for-age under fifth percentile in children and adolescents that is not due to another health condition or the unavailability of food” [3].

Both classification systems introduced different BMI-specifiers to grade the underweight of patients diagnosed with AN. In DSM-5 the subcategorizations for mild, moderate, severe and extreme AN are based on absolute BMI of (i) ≥ 17, (ii) 16–16.99, (iii) 15–15.99 and (iv) < 15 kg/m^2^. For children and adolescents, the corresponding BMI-centiles are to be used (not specifically delineated in the main text). DSM-5 allows for an increase in the level of severity “to reflect clinical symptoms, the degree of functional disability, and the need for supervision” [1]. The ICD-11 subcategorization merely separates adult patients according to BMI ≥ 14 or < 14 kg/m^2^ to define AN with significantly and dangerously low body weight, respectively. For children and adolescents, ICD-11 refers to a BMI below the fifth centile and ≥ 0.3rd centile and a BMI < 0.3rd centile for the specification in these two subcategories [3].

Further research is warranted to describe the BMI-distribution of patients < 18 years in relationship to age. We are aware of a single study that has previously classified young patients with AN and atypical AN upon initial referral to a Danish university eating disorders unit according to low (< 5th centile), medium (5th to 10th centile) and high (≥ 10th centile) BMI-centile groups [4]. The investigators questioned the applicability of the fifth BMI-centile as a substantiated cut-off for the weight criterion in youths with AN due to the fact that 12% and 15% of patients diagnosed as having AN belonged to the medium and high BMI-groups.

The use of the fifth BMI-centile as the weight cut-off had previously been deemed too strict in that it would prevent a larger group of patients from receiving the diagnosis of AN [5, 6]. In addition, the DSM-IV weight cut-off (“body weight less than 85% of that expected”) had been shown to correspond to absolute BMI-values that age dependently fall between the fifth and tenth BMI-centile [2], leading the investigators to favor the use of the tenth BMI-centile as the more inclusive weight cut-off.

The aims of the current study were to descriptively assess the distributions of absolute BMI, BMI-centiles, and BMI-standard deviation scores (BMI-SDS) of young patients requiring inpatient treatment in relationship to age to subsequently assess the DSM-5 and ICD-11 weight criteria and severity specifiers for childhood and adolescent AN. We also compare our results to internationally available studies on DMS-5 severity specifiers. Because starvation induced stunting may affect patients with a younger age at onset more strongly than those with an older age at onset [7, 8], we additionally assessed body height-SDS in relationship to age. In light of largely lacking data on the clinical implications of a severity grading based on body weight at referral, we assessed the relationship between different severity staging criteria and mean length of inpatient treatment and BMI and BMI-SDS at discharge, respectively. We specifically hypothesized that a subgroup of the inpatients diagnosed as having AN has a BMI above the fifth centile.

## Materials and methods

Data of patients of the multi-center German Registry of Children and Adolescents with AN [9–11] were used for the current analyses. Enrolled inpatients were admitted to the 16 participating child and adolescent psychiatric hospitals [10, 11] between August 2014 and February 2019. The ethics committees of all centers approved the registry study. Only complete records of patients including sex, age, date of referral, date of discharge, weight, height (both at referral and discharge) and diagnosis of DSM-5 AN (including subtype) or atypical AN within the umbrella diagnosis ‘Other Specified Feeding or Eating Disorder’ were included in the study. DSM-5 diagnoses were clinical diagnoses by the clinician in charge at each of the local centers. DSM-5 criteria were provided in the registry data entry to increase reliability of the diagnoses; a BMI up to the 10^th^ centile was perceived as still compatible with the DSM-5 weight criterion for AN. Atypical AN was diagnosed if only two of the three criteria for AN were met and the overall clinical impression was considered as being similar to AN (this definition of atypical AN deviates slightly from that given in DSM-5 to additionally allow this diagnosis in patients fulfilling the A criterion and either criterion B or C).

Apart from availability of all data outlined above, the following inclusion criteria were applied to the selection of the respective data sets: female sex (exclusion of 21 males), age < 19 years at referral and written informed consent (patient and legal guardians). Treatment duration was calculated as the difference between dates of discharge and referral (for data protection reasons only months and years were available).

At all participating centers body weight and height were measured at referral and discharge using calibrated hospital scales and stadiometers. Participants were weighed in underwear without shoes. BMI was calculated by dividing weight by the square of height (kg/m^2^). On the basis of nationally representative German reference data for children (KiGGS) [12], individual BMI-values were transformed into BMI-SDS and BMI-centiles using the method suggested by Cole [13]. The method was adapted for the calculation of BMI-SDS by Kromeyer-Hausschild et al. (2001) [14]. The calculation followed the formula: SDS_LMS_ = ([BMI/*M*(*t*)]*L*(*t*) − 1)/(*L*(*t*)*S*(*t*)), with following abbreviations: *L*: Box-cox-power-transformation; *M*: median; *S*: variation coefficient; BMI: individual BMI [14]. The BMI-SDS approximates the deviation of an individual BMI from the median of the reference group expressed in units of the standard deviation.

In ten patients with a BMI ≥ 10th centile, the clinician based diagnosis of AN, restricting type, was converted to atypical anorexia (see discussion). We determined the BMI-SDS corresponding to the ICD-11 specifier (0.3rd centile) using the German reference data [12]. Using the same method [13] and reference data [12], we also transformed individual body heights into height-SDS to investigate potential age dependent effects of starvation induced stunting on body height.

We applied local regression („loess “) to fit smooth curves to some of our scatterplot data with Epanechnikov–Kerner-function using 50% points to fit [15]. The procedure is a fairly direct generalization of traditional least-squares methods for data analysis. The procedure is nonparametric in the sense that the fitting technique does not require an a priori specification of the relationship between the dependent and independent variables. Data analysis with loess allows the exploration of bivariate and multivariate data, assessment of functional forms for relationships among variables, examination of model assumptions in regression analysis, and representation of complex structures within data. The major weakness of loess like of all nonparametric fitting methods is that it cannot be used to characterize the data in terms of a simple equation. The second limitation is that this method requires the analyst to make several partially arbitrary decisions about the fitting parameters [15].

Descriptive data are presented as means, standard deviations, 90% confidence intervals of means (computed using standard error and z-score), ranges, and 5^th^ and 95^th^ percentile. Spearman’s correlations were calculated to assess associations between age and BMI. The confidence intervals for correlation coefficients were computed by the bias corrected and accelerated bootstrap method (BCa 95% CI) [16]. We tested the effect of potential covariates such as centre (linear mixed model), year of admission (ANCOVA) and disease duration (linear regression model) on associations between age and BMI or BMI-SDS.

To compare the patients at time of discharge (*T*1) in relationship to the ICD-11 severity specifier (0.3rd BMI-centile), we performed the Mann–Whitney test.

Exact two-sided significances were calculated, the alpha level was set to 0.05. We performed two separate corrections of P values for multiple testing according to Bonferroni: (1) for 21 correlations (according to our original analysis plan, 21 tests were conducted, of which 7 were not presented in the final manuscript) between age at referral (T0) and BMI and BMI-SDS of inpatients with AN or atypical AN (see Table 2), (2) for three tests to assess the ICD-11 specifier for data at discharge (see Table 4). All analyses were performed using IBM^®^ SPSS^®^ Statistics 25.0.0.1 for Windows. The plots for the Supplementary information were created with R (R Core Team (2018), a language and environment for statistical computing. R Foundation for Statistical Computing, Vienna, Austria, https://www.R-project.org/).

## Results

Descriptive data and the distribution of variables in patient groups diagnosed with AN or atypical AN according to DSM-5 are shown in Table 1 and Fig. S1–S3.

**Table 1:** Descriptive data (mean, SD; 90% confidence interval of mean; range; 5th, 95th percentile) at referral of patients included in the multi-center German Registry of Children and Adolescents with DSM-5 diagnoses of Anorexia Nervosa (AN) (*n* = 404) or Atypical AN (*n* = 65)

	All (*n* = 469)	AN (Restricting type) (*n* = 376)	AN (Binge-eating/purging type) (*n* = 28)	Atypical AN (*n* = 65)
	Mean (SD); [CI 90%]; range; (5th, 95th percentile)			
Age (years)	15.17 (1.64); [15.04,15.29]; 8.92–18.92; (12.04,17.67)	15.13 (1.62); [14.99, 15.27]; 8.92–18.92; (12.93, 17.31)	16.35 (1.20); [15.97, 16.74]; 14.50–18.66; (14.57, 18.59)	14.88 (1.72); [14.52,15.23]; 11.16–17.91; (11.50, 17.59)
Duration of treatment (wks)	17,21 (8.64); [16.55, 17.87]; 0.00–61.00; (4.43, 34.71)	18.01 (8.62); [17.27, 18.74], 0.00–61.00; (4.43, 34.71)	15.39 (10.17); [12.11, 18.66]; 0.00–39.00; (1.8, 37.2)	13.37 (6.88); [11.95,14.80]; 0.00–35.00; (4.29, 26.29)
BMI T0	15.36 (1.53); [15.24, 15.47]; 11.56–20.69; (12.98,17.90)	15.14 (1.34); [15.03, 15.25], 11.56–18.93; (12.93, 17.31)	16.29 (0.99); [15,97, 16,61]; 14.22—17.78; (14.38, 17.78)	16.21 (2.19); [15.76,16.67]; 12.05—20.69; (12.92,19.19)
BMI-SDS T0	− 2.88 (1.19); [− 2.97, − 2.79];− 8.17−(− 0.07); (− 5.03, − 1.21)	− 3.01 (1.12); [− 3.10,− 2.91]; − 8.17-(− 1.29); (− 5.17, − 1.46)	− 2.57 (0.70); [− 2.79, − 2.34]; − 4.49− (− 1.39); (− 4.12, − 1.56)	− 2.29 (1.52); [− 2.60,− 1.97]; − 6.27− (− 0.07); (− 5.24, − 0.51)
BMI-centile T0	2.32 (5.13); [1.93, 2.71]; 0.00–47.11; (0.00,11.24)	1.28 (2.24); [1.09;1.47]; 0.00–9.79; (0.00, 7.18)	1.37 (1.79); [0.79, 1.94]; 0.00—8.21; (0.01, 6.26)	8.71 (10.64); [6.51,10.91]; 0.00—47.11; (0.00, 30.61)
BMI T1	18.29 (1.43); [18.18,18.40]; 13.63–22.90; (15.78, 20.34)	18.29 (1.41); [18.17,18.41]; 13.63–22.90; (15.83, 20.31)	18.63 (1.31); [18.20,19.05]; 15.86—20.72; (16.18, 20.61)	18.18 (1.59); [17.85,18.51]; 14.39—20.87; (15.35,20.71)
BMI-SDS T1	− 1.14 (0.76); [− 1.20,− 1.08]; − 6.14–0.86; (− 2.68, − 0.24)	− 1.14 (0.76); [− 1.20, − 1.07]; − 6.14–0.86; (− 2.63, − 0.27)	− 1.23 (0.70); [− 1.46, − 1.00]; − 3.31− (− 0.23); (− 3.03, − 0.26)	− 1.11 (0.84); [− 1.29, − 0.94]; − 4.31–0.08; (− 2.83, − 0.20)
BMI-centile T1	17.60 (12.78); [16.62,18.57]; 0.00–80.43; (0.36, 40.62)	17.47 (12.62); [16.39,18.54]; 0.00–80.43; (0.42, 39.52)	15.14 (10.93); [11.62, 18,66]; 0.05—40.89; (0.19, 39.89)	19.41 (14.31); (0.00–53.35); [16.45;22.37]; (0.24,42.21)

*SD* standard deviation, *wks* weeks, *CI* confidence interval of mean, *T0* referral, *T1* discharge

### Relationship between age and BMI and BMI-SDS

**Fig. 1:**
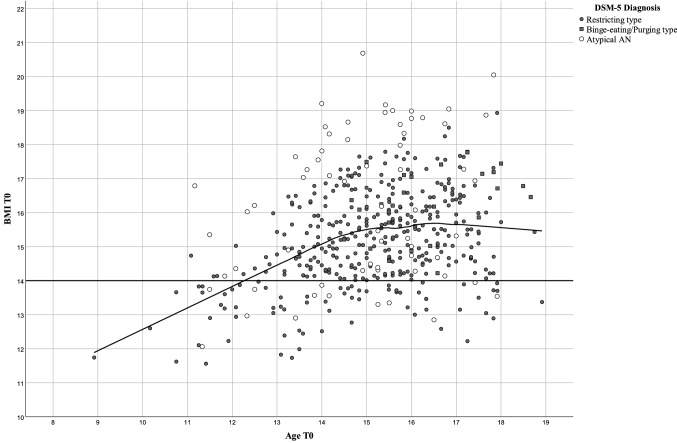
Scatterplot for age and BMI at referral (T0) of 469 inpatients with Anorexia Nervosa (AN; differentiated according to restricting or binge eating/purging type) or atypical AN including local regression (loess) in relationship to the ICD-11 specifier of BMI ≥ 14 or < 14 kg/m^2^ (straight line)

**Fig. 2:**
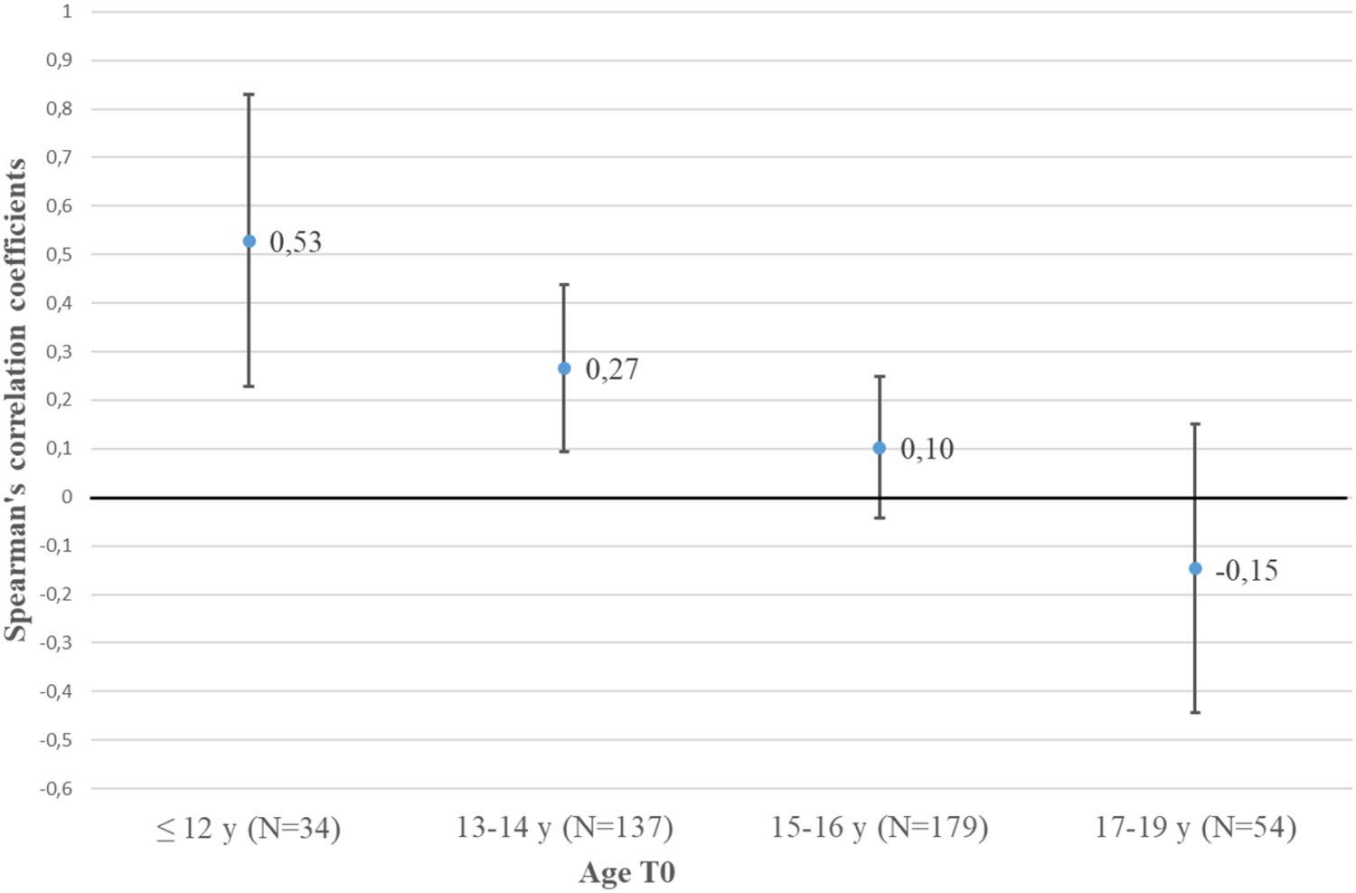
Age dependency of mean correlations (Spearman’s) and 95% confidence intervals between age and BMI at referral (T0) of 404 inpatients with Anorexia Nervosa for four age groups. Correlation coefficients were calculated on a cross-sectional basis per age group

The mean age of the 469 patients (AN + atypical AN) was 15.17 (SD = 1.64; range 8.92–18.92) years. At the descriptive level BMI at referral increased to age 15 to subsequently plateau up to age 18 (Fig. 1), which represented the oldest age for study inclusion. Correlations between BMI and age (Table 2) confirmed the visual impression: Only BMI of patients < 15 years (*n* = 200; 42.6% of all patients) moderately increased with age (*r* ≈ 0.5); in contrast, the sub-analysis of patients aged ≥ 15.0 years revealed a correlation close to zero. Based on our cross-sectional data, correlations within age defined subgroups including the 95% confidence intervals (CI) between BMI and age were highest in the youngest age group of the 404 patients with AN; as of age 15 the 95% CI included zero (Fig. 2).

**Table 2:** Correlations between age at referral (T0) and BMI and BMI-SDS of inpatients with Anorexia Nervosa (AN) or atypical AN

	All (*n* = 469)	AN (*n* = 404)	Atypical AN (*n* = 65)	AN < 15 years (*n* = 171)	Atypical AN < 15 years (*n* = 29)	AN ≥ 15 years (*n* = 233)	Atypical AN ≥ 15 years (*n* = 36)
*BMI kg/m*^*2*^							
*r*	0.28*	0.32*	0.24	0.47*	0.54	0.03	0.09
*P*	5.2 × 10^–13^	3.5 × 10^–11^	0.058	1.19 × 10^–10^	0.003	0.618	0.597
CI	[0.19, 0.37]	[0.23, 0.41]	[− 0.02, .46]	[0.33, 0.59]	[0.23, 0.77]	[− 0.11, 0.18]	[− 0.29, 0.43]
BMI-SDS							
*r*	− 0.30*	− 0.31*	− 0.21	0.02	0.28	− 0.26*	− 0.06
*P*	2.3 × 10^–11^	1.15 × 10^–10^	0.099	0.842	0.146	4.9 × 10^–5^	0.714
CI	[− 0.39, − 0.21]	[− 0.39, − 0.22]	[− 0.44, 0.04]	[− 0.15, 0.18]	[− 0.12, 0.64]	[− 0.39, − 0.13]	[− 0.42, 0.28]

*r* correlation coefficient (Spearman’s rho), *p* *P* value, *CI: 95%* confidence intervals computed by bias corrected and accelerated bootstrap method, *y* years

*Significant after Bonferroni correction (*P* < 2.38 × 10^–3^)

**Fig. 3:**
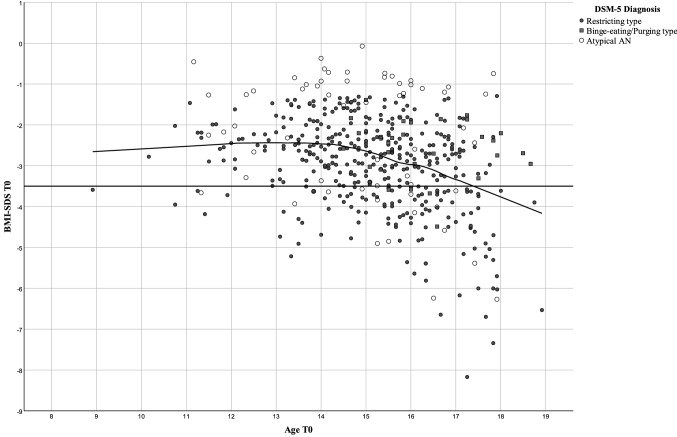
Scatterplot for age and BMI-SDS at referral (T0) of 469 inpatients with Anorexia Nervosa (AN; differentiated according to restricting or binge eating/purging type) or atypical AN including local regression in relationship to the ICD-11 specifier of BMI-centile ≥ 0.3 or < 0.3, which corresponded to BMI-SDS across the age range that skewed between 3.40 and 3.48

In contrast to absolute BMI, BMI-SDS was negatively correlated with age at referral for inpatient treatment (Table 2; Fig. 3), i.e., older patients at referral tended to have a BMI more deviant from the normal range of their age group than younger patients. The negative correlation was only accounted for by patients aged 15 or older. The analyses pertaining to the potential covariates revealed no significant effect of center, year of admission or duration of the eating disorder (data not shown; for descriptive data see Figs. S4–S7, Tables S1 and S2).

The transformation of BMI-SDS into BMI-centiles eliminated a substantial part of the variance in this specific patient group (Figs. S1c, S3c). Thus, 81 patients had a BMI < 0.01 centile (corresponding to a BMI-SDS < – 3.9), entailing a clustering at the centile of zero.

**Fig. 4:**
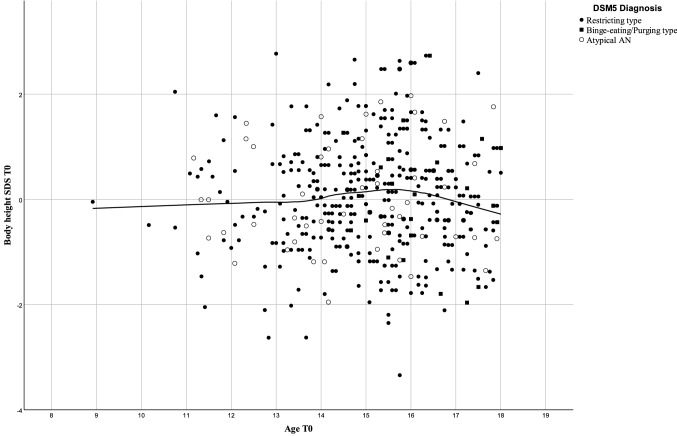
Scatterplot for age and body height-SDS of 469 inpatients with Anorexia Nervosa including local regression (loess). Linear correlation *r* = 0.009, *P* = 0.844

We found no relationship between age at referral and height-SDS (Fig. 4).

### Weight criterion

Of the 404 patients with AN (restricting type *n* = 376 and binge eating/purging type *n* = 28) *n* = 361 (89.4%) had a BMI < 5th centile (Table 3).

**Table 3:** Numbers and percentages of inpatients fulfilling the recommended DSM-5 and absolute ICD-11 weight criterion for Anorexia Nervosa (AN) based on the fifth BMI-centile

BMI-centile	All patients (*n* = 469)	AN restricting type (*n* = 376)	AN binge-eating/purging type (*n* = 28)	Atypical AN (*n* = 65)
< 5	395 (84.2%)	334 (88.8%)	27 (96.4%)	34 (52.3%)
≥ 5	74 (15.8%)	42 (11. 2%)	1 (3.6%)	31 (47.7%)

### Severity specifiers

One-half of the 404 patients with AN fulfilled the *childhood and adolescent* ICD-11 specifications for significantly (BMI-centile ≥ 0.3) and dangerously (BMI-centile < 0.3) low body weight, respectively (Table 4). Compared to patients with significantly low BMI, those with dangerously low BMI had a 2-week longer mean treatment duration (Mann–Whitney *U* = 17,128.5; *P* = 0.005), 0.46 kg/m^2^ lower BMI at discharge (*U* = 16,992.5; *P* = 0.004) and 0.44 lower BMI-SDS at discharge (*U* = 13,942.5; *P* = 3.7 × 10^–8^). All three *P* values were lower than the Bonferroni corrected p value of 0.017.

**Table 4:** Inpatients with Anorexia Nervosa fulfilling the ICD-11 specifications of significantly low and dangerously low body weight at referral (T0) in relationship to mean inpatient treatment duration and BMI and BMI-SDS at discharge (T1)

ICD-11 BMI-centile	*n*	Mean treatment duration in weeks (± SD)	BMI T1 mean (± SD)	BMI-SDS T1 mean (± SD)
≤ 0.3	202	18.78 (8.43)	18.08 (1.54)	− 1.36 (0.88)
> 0.3	202	16.85 (8.97)	18.54 (1.22)	− 0.93 (0.52)

Because BMI revealed no age dependency in adolescents aged 15–18 (Fig. 1, Table 2), we descriptively assessed both the DSM-5 and ICD-11 *adult* severity specifiers based on absolute BMI-cut-offs (Table 5 and Table 6) and used inpatient treatment duration and both BMI and BMI-SDS at discharge as proxies to assess the clinical validity of the different severity categories.

**Table 5:** DSM-5 severity specifiers based on absolute BMI-values at referral (T0) according to DSM-5 in patients with Anorexia Nervosa (AN) aged ≥ 15 years and relationship with BMI, BMI-SDS at discharge (T1) and treatment duration

BMI T0	AN (*n* = 233)	BMI T1 mean (SD)	BMI-SDS T1 mean (SD)	Treatment duration (weeks)
< 15 kg/m^2^	81 (34.76%)	18.17 (1.47)	− 1.59 (1.03)	20.57 (9.57)
15–15.99 kg/m^2^	66 (28.33%)	18.74 (1.41)	− 1.20 (0.79)	16.94 (7.39)
16–16.99 kg/m^2^	57 (24.46%)	18.93 (1.09)	− 1.09 (0.55)	16.32 (8.66)
≥ 17 kg/m^2^	29 (12.45%)	19.34 (0.92)	− 0.91 (0.42)	11.27 (5.53)

**Table 6:** ICD-11 adult specifiers for significantly low and dangerously low body weight based on a BMI < or ≥ 14 kg/m^2^ at referral (T0), respective absolute BMI and BMI-SDS at referral for inpatient treatment in 233 patients with Anorexia Nervosa aged ≥ 15 years and relationship with BMI, BMI-SDS at discharge (T1) and with treatment duration

BMI at referral	*N*	BMI T0 mean (SD)	BMI-SDS T0 mean (SD)	BMI T1 mean (SD)	BMI-SDS T1 mean (SD)	Treatment duration mean (SD)
< 14 kg/m^2^	23 (9.9%)	13.41 (0.45)	− 5.66 (0.99)	17.88 (1.91)	− 1.93 (1.48)	21.93 (11.51)
≥ 14 kg/m^2^	210 (90.1%)	15.79 (1.07)	− 2.98 (0.87)	18.75 (1.26)	− 1.20 (0.70)	16.84 (8.32)

## Discussion

Both BMI and BMI-SDS at referral are clearly age dependent in young inpatients with acute AN, with the correlations being positive for BMI and negative for BMI-SDS. With respect to absolute BMI, females aged < 15 years as a group tended to have lower referral BMI than patients ≥ 15 years old. Our sample included 34 patients aged < 12 years, for whom we found the highest correlation (*r* ≈ 0.5; Fig. 2) between age and BMI; this correlation drops in the next oldest age groups to approach zero as of age 15 (Table 2). It deserves mentioning that the absolute BMI-values constituting the fifth or tenth BMI-centile according to both the US CDC [17] and German KiGGS [12] reference data do not plateau, thus excluding the possibility that the observed plateau in inpatients with AN is related to a general cessation of increments in BMI in the underweight range between ages 15 and 18.

It is of obvious interest to determine if the plateau observed between age 15 and 18 persists into adulthood (in particular during the age span during which AN manifests) or if further age dependent increments occur after age 18. If the plateau indeed extends beyond age 18, both weight cut-offs for the diagnosis of AN and absolute BMI-severity specifiers could apply as of age 15, thus allowing the use of the same cut-offs/specifiers in adults and adolescents aged ≥ 15 years. Because, in younger patients, BMI-SDS shows no correlation to age (Table 2), a specific BMI-SDS or BMI-centile (e.g., 10th BMI-centile; see below) could be used as the weight criterion.

The drop in correlations between BMI and age (Fig. 2) presumably reflects the increasing number of female patients who completed puberty. Thus, in a longitudinal study including 615 white females recruited at age 9 (77.2% pre-pubertal) and followed-up for 10 years the mean onset of puberty was 10.2 years, being 12.6 years for the onset of menarche and 14.3 years for entering Tanner growth stage 5 [18]. Full adult height was achieved at 17.1 years. The correlations between age and BMI at referral dropped across the time span during which puberty and also the relative increase in fat mass occurs.

We hypothesize that the negative correlation between age and BMI-SDS reflects the increasing fat mass and % body fat (total fat mass divided by total body mass × 100; %BF) in females during puberty. During puberty, % body fat increases by approximately 50% in females [19]. As such, older females may be able to lose more weight due to a higher fat mass before fat free mass is affected to such a degree that health is seriously compromised. Thus, lower BMI-SDS may be achieved in older individuals. Indeed, the comparison of body composition prior to and after weight gain in 130 patients with AN with a mean age of 20 years revealed a stronger increment in mean fat mass (6.6 kg) than in mean fat-free mass (5.2 kg) [20].

Starvation induced stunting should preferentially affect those patients who have not achieved their final height. Stunting should entail disproportionately higher BMI in younger patients due to the effect of a reduced squared height in meters as the denominator of the index. However, we observed the opposite: we found lower BMI in young patients as compared to those aged > 15 years. Further, height-SDS showed no relationship to age (Fig. 4). Anorexia nervosa seemingly does not affect growth to an extent that would allow its detection in our large sample including 171 patients aged < 15 years. In accordance with our results, two recent meta-analyses found no deviation in height at baseline [7, 21]. Nevertheless, stunting has repeatedly been reported in patients with AN [7]. Apparently, the relationship between stunting and starvation is more complex and seemingly cannot be reduced to nutrition alone [22].

In clinical practice, BMI-centiles are used to define cut-offs for different weight categories [1, 3]. The DSM-5 weight recommendations were based on CDC reference BMI data [1]. International comparisons of absolute BMI-values constituting specific centiles in the underweight range are warranted and need to be set into relationship to representative data sets for referral BMI of patients with AN. Because the absolute BMI-values constituting the 10^th^ BMI-centile in both CDC and KiGGS data sets reveal slight differences which increase with age, the use of the 10^th^ BMI-centile as weight cut-off in both the US and German population would entail slightly higher absolute BMI-values in the German data set (likely the result of different socio-economic, ethnic and ancestry compositions of respective populations) [12, 17].

Irrespective of these differences, this study again underscores that the BMI-cut-off based on the 5^th^ centile provided in both DSM-5 (main text) and ICD-11 for children and adolescents is too strict entailing that a substantial proportion of patients with an AN-like phenotype would need to be classified as atypical AN. This situation is exactly opposite to one of the central aims of the DSM-5 Eating Disorders Work Group, namely the reduction of the percentage of eating disordered patients with a DSM-IV diagnosis of an Eating Disorder Not Otherwise Specified [23]. Notably, in DSM-5 the fifth BMI-centile is provided merely as a guideline, whereas in ICD-11 this cut-off is an integral part of the diagnostic criteria. Accordingly, it is particularly the strict ICD-11 weight criterion that in our opinion requires reconsideration. If it is to remain a diagnostic feature, we would have diagnosed atypical AN in 10.6% of our patients (all patients with a BMI between the fifth and tenth centile) despite a clinical symptomatology otherwise indistinguishable from AN. In a recent Danish study [4] 12% of the patients diagnosed (ICD-10 criteria) as having AN had a referral BMI between the fifth and tenth BMI centile, another 15% out of the total of 182 patients with AN had a BMI ≥ 10th centile. The larger percentage in the Danish study as compared to our own is in part due to our decision to not allow for the diagnosis of AN, if the BMI of a patient was ≥ 10th centile; it may also reflect the inclusion of both outpatients and inpatients (the setting is not specified) by Andersen et al.

ICD-11 could thus refer to a more descriptive weight criterion as in DSM-5. However, the disadvantage of a descriptive and non-fixed weight criterion is that the subjective evaluation of “a significantly low body weight in the context of age, sex, developmental trajectory, and physical health” [1] renders the comparison of clinical and epidemiological data difficult. Overall, the hesitancy to provide a weight cut-off in the A criterion of DSM-5 is in our opinion not justified. If underweight is to remain a clinical feature of AN, then a rather high centile at the boundary between the underweight and normal weight range would imply that the A criterion can be endorsed in most patients with an AN-like symptomatology. For the purpose of this study, we had converted all diagnoses of AN to atypical AN, if the BMI exceeded the tenth centile, which we considered as the uppermost BMI-cut-off compatible with the DSM-5 A criterion. This approach is obviously debatable but serves to illustrate the difficulties encountered upon the use of a vaguely defined weight criterion, which leaves room for a subjective interpretation by the diagnostician. Hebebrand and Bulik [5] have previously discussed that the weight criterion could be omitted all together from the diagnostic criteria, if it is substituted by the requirement of symptoms of starvation, which at an individual level would be compatible with “a significantly low body weight in the context of age, sex, developmental trajectory, and physical health” [1].

Transition also merits consideration in that the childhood/adolescent weight cut-off should be continuous with that for adults. In this context, it deserves to be pointed out that according to US and German data [12, 17] BMI values of 18.19 kg/m^2^ and 18.95 kg/m^2^, respectively, correspond to the 10th centile in females at age 18.0. Accordingly, the use of the 5th centile for children and adolescents would entail a considerably stricter threshold for the weight criterion in AN than in adults; the use of the 10th percentile would allow for a rather smooth transition. As delineated above, the DSM-5 and ICD-11 adult weight cut-off (BMI < 18.5 kg/m^2^) might already be applicable as of age 15. Based on the CDC (KiGGS) growth charts [12, 17] a BMI of 18.5 kg/m^2^ corresponds to BMI-centiles of 30 (18), 23 (13), 17 (9) and 13 (approx.6) at ages 15.0, 16.0, 17.0 and 18.0, respectively.

Three issues are relevant with respect to the severity specifiers for AN. (1) The percentages of patients fulfilling particular specifiers requires clarification. (2) The specifiers for childhood/adolescent AN should in essence capture similar percentages of patients as in adulthood; this is particularly pertinent upon the transition into adulthood. (3) Finally, the specifiers should have clinical validity.

### Percentages of patients fulfilling particular specifiers

Based on our large sample of inpatients with AN, we were able to show that the ICD-11 childhood and adolescent severity specifier (0.3rd BMI-centile) divided our sample into two equally large groups.

### Specifiers and transition

Because the proportion of 15–18 year-old patients who fulfilled the ICD-11 adult specification of a dangerously low body weight (BMI < 14 kg/m^2^) was only 9.9% (Table 6) and therefore substantially lower than when using the 0.3rd BMI-centile (50%; Table 4), the two cut-offs are not compatible. Thus, the severity specifier for children and adolescents is much more lenient than for adults entailing that upon transition into adulthood the two severity specifiers do not capture a similar group and percentage of patients. This discordance obfuscates comparability of predictor research of outcomes in youth and in adults, and a desirable harmonization of severity thresholds should be attempted. Future research is required to assess if the adult severity specifiers can already be used as of age 15 (Tables 5 and 6), as our data suggest. As such, comparisons of the percentages of patients with AN, who fulfill the BMI-criteria for the severity specifiers is warranted. Such a comparison is currently only possible with respect to the DSM-5 specifiers (we are unaware of studies that have assessed the severity specifiers provided for young and adult patients in ICD-11): Among 128 adult patients treated at an Italian hospital 32.0%, 15.6%, 14.1% and 38.3% were classified as having mild, moderate, severe and extreme AN, respectively [24]. In a second Italian study, 273 patients with AN were drawn from a sample of 1647 adults of both sexes consecutively referred to and assessed for treatment of an eating disorder at three medium size specialized treatment centres/sites [25]. The percentages of patients assigned to each DSM-5 category were rather similar and ranged from 23.1% (mild) to 26.7% (severe). No significant differences in current age and age at onset of AN were observed between these categories. In a Danish clinical sample of 146 adult patients including six males, frequencies were 23.3% (mild), 24.0% (moderate), 21.9% (severe) and 30.8% (extreme) [26]. In a Portuguese sample of 201 (including 8 males) treatment-seeking patients with a mean age of 22.4 years (SD = 9.5; range = 11–61 years), the respective frequencies were 36.3%, 19.9%, 14.9% and 28.9% [27]; again, no significant age differences were observed between the four groups. Finally, among 109 adult females with AN, who had initially been phone screened for eligibility and who consented to participate in a research project, the frequencies for mild and extreme severity of AN were 64.2% and 3.7%, respectively [28]. A look at the percentage of our inpatients with AN aged ≥ 15 years, who fulfill the extreme severity specifier according to DSM-5 (34.8%; see Table 5), indicates that this proportion is within the range of the respective percentages reported in adult patients. Our inpatient sample based on registry data from 16 hospitals is to our knowledge the largest, in which both the ICD-11 and DSM-5 specifiers have been investigated.

Whereas this limited amount of data indicates that there may be no major differences of the frequencies of the four severity grades between adults and adolescents aged 15–18 and in particular in the extreme category, a systematic international approach combining clinical data from young and adult patients is warranted to further investigate this important issue. A population-based approach would exclude the difficulties with respect to representativeness inherent to clinical outpatient or inpatient samples; however, the number of identified patients will be low even in large epidemiological samples. Nevertheless, such data if based on a sufficiently large sample might offer an orientation. We are aware of a single epidemiological study, in which a small number (*n* = 16; including one male) of AN patients were identified [29]. According to the authors the minimum level of severity was based on BMI (for adults; for children and adolescents on BMI percentiles), and may be increased to reflect clinical symptoms, the degree of functional disability, and the need for supervision” [29]. The respective BMI-centiles for the categorization of severity were not provided, but five, four, four and three patients were categorized as mild, moderate, severe, and extreme.

*Validity of specifiers* The clinical validity of the four DSM-5 severity categories has been questioned. Most [24–27, 30], but not all [29] studies have questioned the validity of these specifiers using different approaches based on age [26], eating disorder symptomatology including binge eating, purging behavior and excessive exercise [24, 26–28, 30], comorbid psychopathology [28], outcome (weight recovery, good outcome) [24], number of previous hospitalizations [30], quality of life, physical health, psychosocial impairment, and seeking of treatment by health care services [29]. Collectively, these data seem to support that the severity criteria need to be revised or, at least, validated in sufficiently large and representative samples, using illness characteristics and outcomes that have sufficient face validity and clinical importance. This conclusion is supported by the results of this study. We have used our registry based data to assess BMI, BMI-SDS and treatment duration in relationship to the DSM-5 and ICD-11 severity specifiers. The differences between severity categories were significant, but not to an extent that they would in our opinion help in daily clinical practice to stratify risk assessment or treatment (Tables 5 and 6).

In children and adolescents, but maybe in adults, too, the interpretation of both the weight criteria and the severity specifiers for AN hinges on the knowledge of the relationship between premorbid BMI and BMI at referral. Significant correlations have been observed between premorbid BMI-centile and BMI at referral in childhood and adolescent patients with AN [31, 32], but we are not aware of a similar study in adults. It is safe to conclude that for children and adolescents with AN, premorbid body weight impinges on both the percentages of patients who have a BMI < 5th centile and who fulfill severity specifiers based on BMI.

Results of this study need to be interpreted within its limitations. Despite the fact that 16 centers contributed to the Registry we cannot reliably deduce that the data are fully representative of German female inpatients with AN. Based on those 14 centers which entered ≥ 10 patients into the Registry, we were unable to detect any centre effect on our results. Replications in independent samples are required to confirm the plateauing of the BMI-distribution as of age 15; the transition into adulthood requires further investigation. Because our Registry does not include data on outpatients, all results need to be interpreted accordingly. We would assume that our inpatients have a lower BMI at referral than the outpatients treated at the same centers because inpatients are admitted at a low weight relative to their individual weight history. Our criticism of the use of the 5th BMI-centile as being too strict would likely be even more valid if outpatients had been analyzed in a similar manner. As pointed out above, a Danish study based on presumably both outpatients and inpatients [4] identified a larger percentage of patients with BMI above this centile in comparison to our data. Currently, we cannot state that the relationship between age and BMI/BMI-SDS applies to outpatients (or a combined sample), too.

Another limitation pertains to the exclusion of males with AN, but the sample of males was too small to allow for meaningful analyses. However, males with AN also need to be studied to assess if the data obtained in females generalize to males too. Another limitation of our study pertains to our inability to assess the exact treatment duration in days due to data protection reasons. We refrained from determining BMI-centile cut-offs for children and adolescents as based on the provision of absolute BMI-values for the severity categories in DSM-5 in light of the need of more data to determine if the adult specifiers are indeed age independent. Nevertheless, despite these limitations, to our knowledge, this is the largest study of children and adolescents with AN investigating the trajectory of BMI, BMI-centiles, and BMI-SDS over the pediatric age range until age 18 and in relationship to DSM-5 severity specifiers, and the only such study that investigated the same question relative to ICD-11 specifiers. As such, we consider the results relevant, as they suggest the potential to use BMI as of age 15 and not 18, at least in females with AN, and as they cast considerable doubt on the validity of the currently conceived BMI-centile cut-off for AN as well as on the severity criteria for AN in youth, introducing significantly different subgroups in youths vs. adults. We to our knowledge for the first time assessed height-SDS in relationship to age.

In conclusion, we found evidence for a plateauing of the BMI-distribution upon attainment of age 15 in female inpatients with AN, suggesting that adult cut-offs based on absolute BMI-values may already be used as of age 15. We found no evidence for stunting in younger patients with AN. The fifth BMI-centile is not suited as the weight criterion for AN, its strict application as delineated in ICD-11 would entail that a considerable subgroup of patients with an AN-like phenotype is not diagnosed as having AN. Finally, the severity specifiers assessed via different approaches have not proven to be of a sufficiently convincing clinical validity; further research is required to attempt to define clinically relevant variables that justify the determination of such severity specifiers.

## Electronic supplementary material

Below is the link to the electronic supplementary material.

Supplementary file1 (PDF 197 kb)
