# Supplementary material for: Age dependency of body mass index distribution in childhood and adolescent inpatients with anorexia nervosa with a focus on DSM-5 and ICD-11 weight criteria and severity specifiers
Source: Eur Child Adolesc Psychiatry. 2020 Jul 14;30(7):1081–94. doi: 10.1007/s00787-020-01595-4 (PMC8295075; doi:10.1007/s00787-020-01595-4)
Supplement: Supplementary file 1 — Supplementary file1 (PDF 197 kb) [file 787_2020_1595_MOESM1_ESM.pdf]

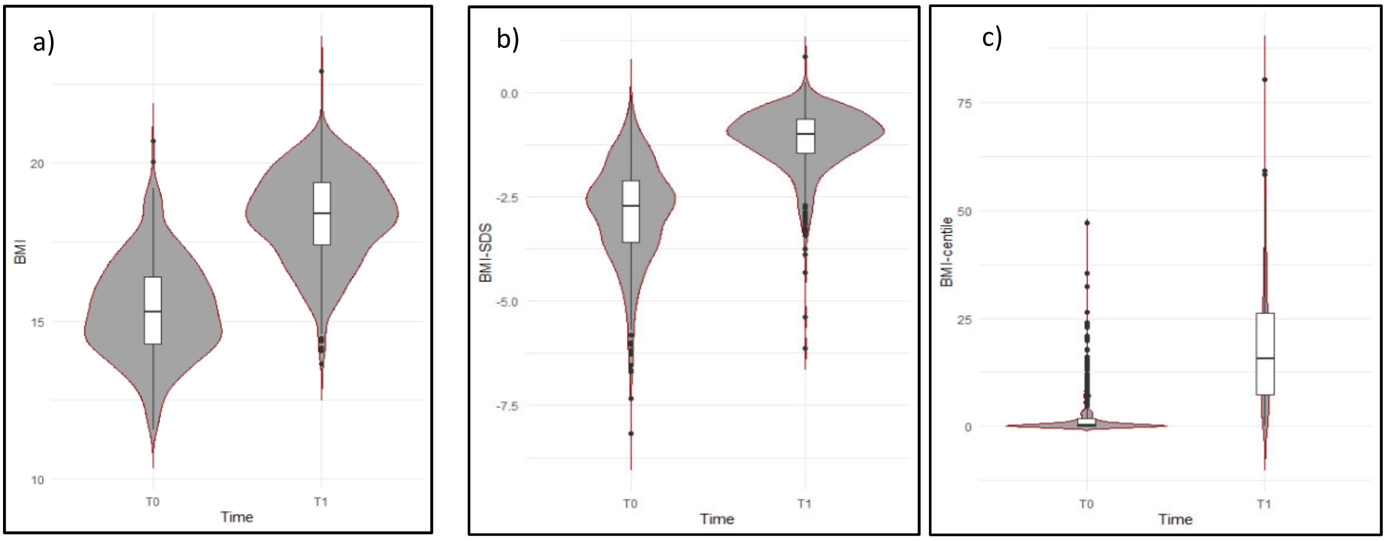

Figure S1. Distribution and probability density of (a) BMI, (b) BMI-SDS and (c) BMI-centiles at T0 and T1 visualized with violin plots (n=469)

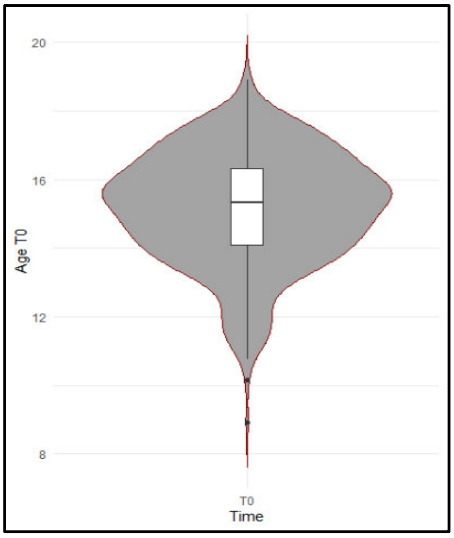

Figure S2. Distribution and probability density of age at T0 visualized with violin plots (n=469)

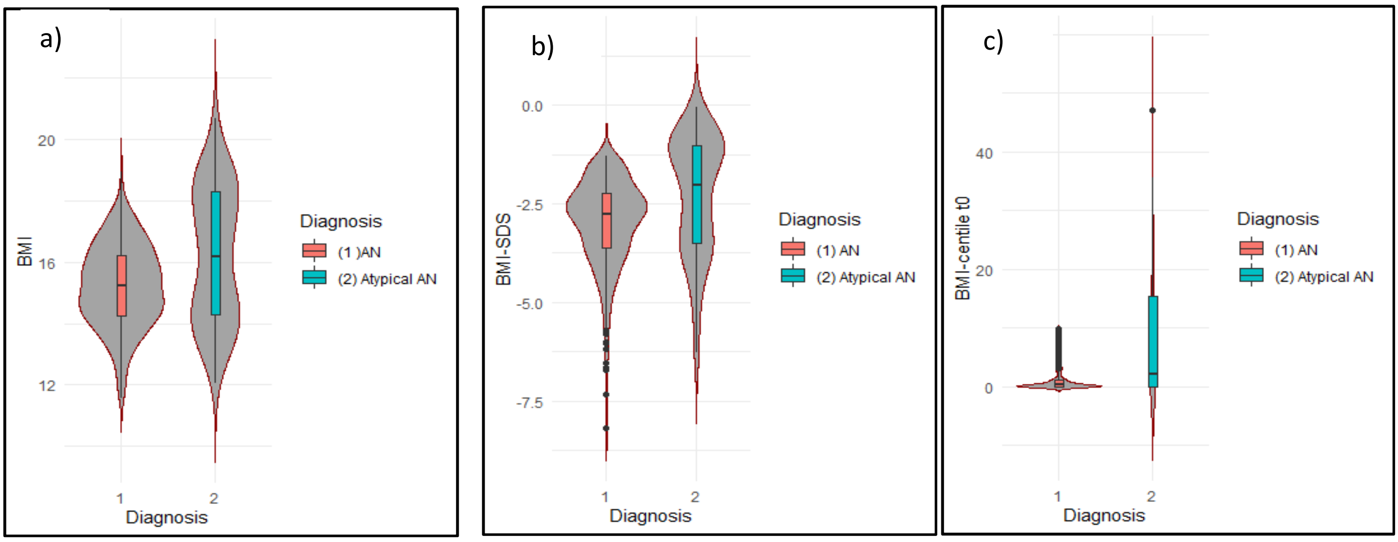

Figure S3. Distribution and probability density of (a) BMI, (b) BMI-SDS and (c) BMI-centile at T0 separated according to the diagnoses of AN (n=40) and atypical AN (n=65) visualized with violin plots

Year	N
2014	22
2015	119
2016	126
2017	116
2018	84
2019	2
All	469

Table S1. Number of patients per year of admission

Centre	N
Aachen	52
Essen	46
Ulm	10
Berlin	35
Bonn	4
Freiburg	33
Würzburg	33
Marburg	44
Homburg/Saar	15
Hamm	24
Tübingen	8
Dresden	91
Viersen	20
Heidelberg	20
Münster	19
Mainz	15
All	469

Table S2. Number of patients per centre

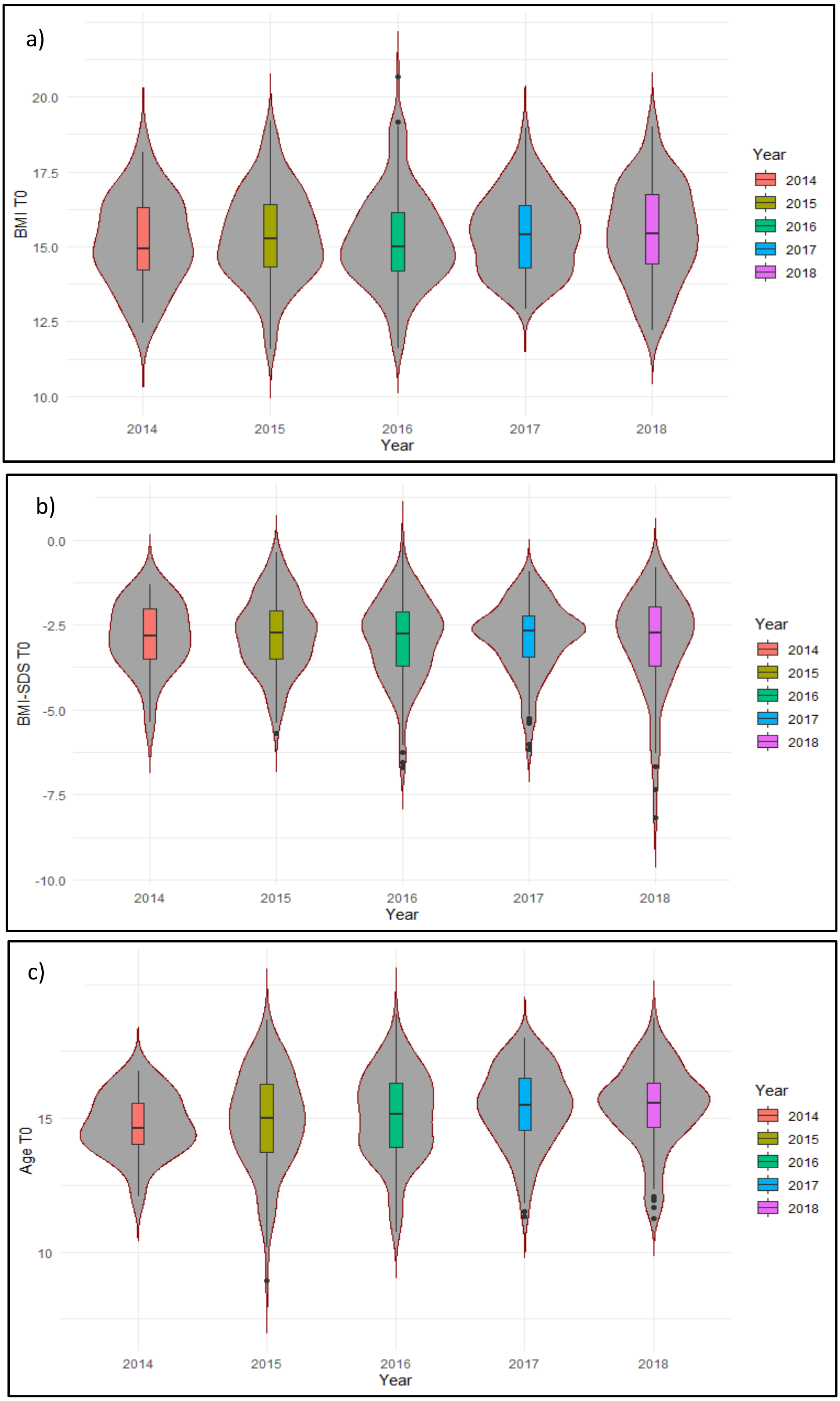

Figure S4. Distribution and probability density of a) BMI, b) BMI-SDS and c) age at T0 according to admission year visualized with violin plots

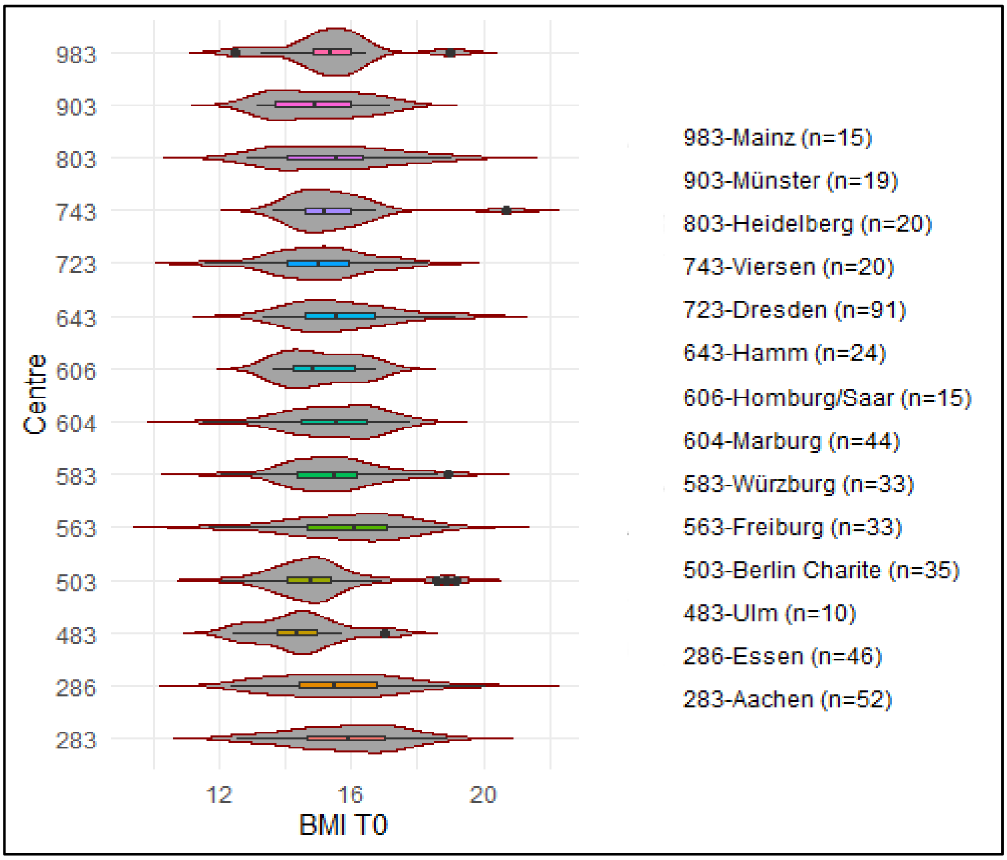

Figure S5. Distribution and probability density of BMI at T0 per centre visualized with violin plots

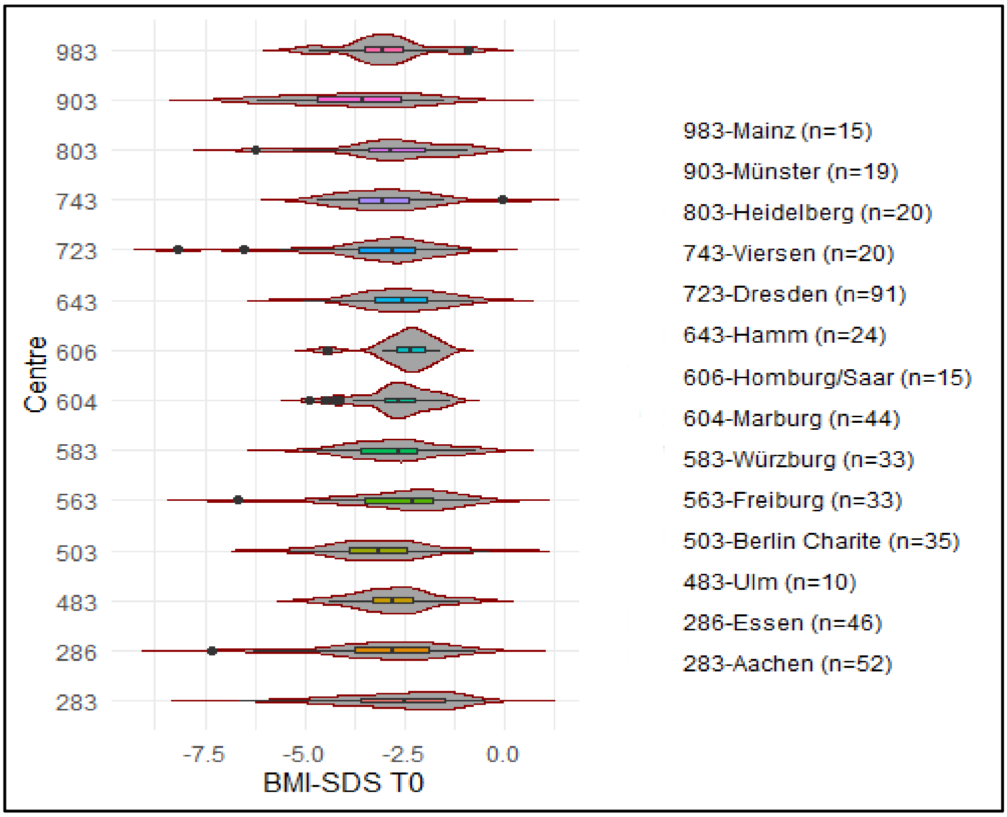

Figure S6. Distribution and probability density of BMI-SDS at T0 per centre visualized with violin plots

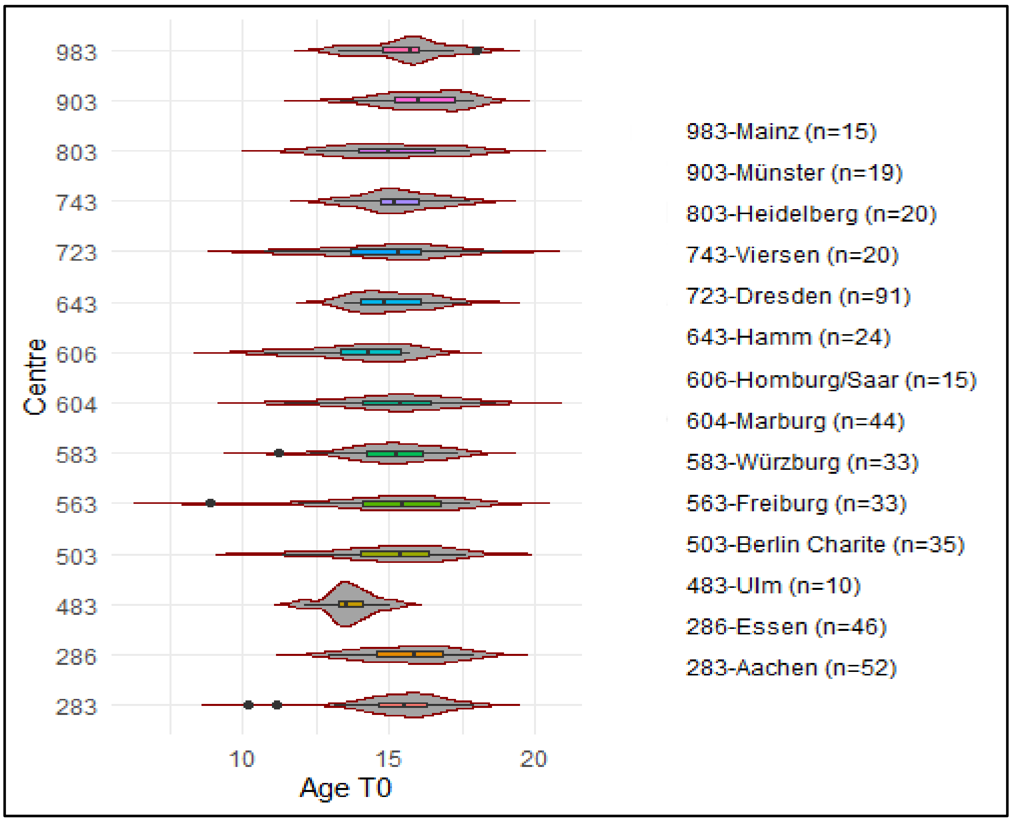

Figure S7. Distribution and probability density of age at T0 per centre visualized with violin plots
